# Editorial: Advances in new combinational therapies for treatment of MDR pathogens

**DOI:** 10.3389/fcimb.2025.1709379

**Published:** 2025-11-04

**Authors:** Sanjit Kumar, Biswajit Mishra, Anshul Chaudhary, Sanket Kaushik

**Affiliations:** ^1^ Guru Ghasidas Vishwavidyalaya, Bilaspur, India; ^2^ Brown University, Providence, RI, United States; ^3^ University of California, San Diego, La Jolla, CA, United States; ^4^ Amity University Rajasthan, Jaipur, India

**Keywords:** MDR, combinational therapeutic strategy, nanoparticle, synergistic effect, ESKAPE bacteria

Multidrug-resistant (MDR) pathogens pose an urgent challenge due to genetic change, misuse of antibiotics, and biofilm production, working against standard treatments. To seek solutions, recent progress has focused on combinational approaches that combine multiple methodologies for greater effectiveness (Elshobary et al., 2025; Kain et al., 2025). Nanotechnology-based delivery systems offer improvements in drug stability and targeting, while natural products offer potential synergistic efficacy with currently available antibiotics (Islam et al., 2025). Treatments are also evolving with photodynamic therapy and immunotherapeutic approaches, as well as the new use of computational drug design and epitope-based vaccines (Haldiya et al., 2024; Thiruppathi et al., 2024). In so doing, these combined or integrated innovations together comprise a unified response to the issues surrounding the impending MDR crisis globally.

This Research Topic aims to give a concise overview of new combinational strategies for treatment of MDR pathogens. By integrating natural products, nanotechnology, computational screening, and vaccine design, the goal is to highlight promising interventions that not only combat resistant pathogens but also enhance therapeutic outcomes and minimize resistance development.

Tanu et al. in a mini review, highlight how photodynamic therapy (PDT), uses a photosensitizer, light and oxygen to produce reactive oxygen species (ROS), has the potential to address already established resistance mechanisms to treatment. PDT is a treatment modality that proves broadly effective against a variety of pathogens *in vivo* due to its ability to penetrate biofilms, generate an immune response, and produce local antimicrobial effects while being considered overall quite safe. Recent advances in antimicrobial blue light and the introduction of next-generation photosensitizers lend support to the PDT approach for the treatment of infectious diseases.

There are several studies that have conducted primary research and reported the use of nanotechnology for the treatment of MDR pathogens. For example, a SabiWhite-loaded ethosome-based topical delivery system was reported to have a vesicle size, entrapment efficiency and zeta potential of 184.4nm, 92.5% and -13.50mV, respectively. The efficacy of the delivery system was optimized to show sustained release (93.12% over 24 hrs) and demonstrated efficacies of 36.17% *in-vivo* edema reduction, relative to Diclofenac gel (41.92%) and no evidence of irritation was observed after 120 days of storage (Shi et al.). Once again, garlic derived AgNPs (15–20 nm), had strong antimicrobial activity against a MDR pathogen, *Escherichia fergusonii*, which has been described as a relevant clinical pathogen and has a 24% prevalence in wound infections, Furthermore, AgNPs produced inhibition zone findings of 28 ± 0.5 mm, a minimum inhibitory concentration (MIC) of 100 mg/mL and compromised the bacterial cell membrane through oxidative stress. The synergy assays showed strong interactions with the antibiotic, ciprofloxacin (FIC index = 0.37) and time-kill assays indicated that bacteria were cleared rapidly by the AgNP when used in combination (Abdelkhalig et al.).

Natural resources also showed the ability to treat diseases. Extracts from the desert plant Indian Borage showed strong antimicrobial, antioxidant (IC50 = 71.97 µg/mL) and anti-inflammatory properties. Interestingly, when mixed with amikacin, over 50% of resistant isolates became sensitive. In mouse models, the combination of amikacin and extract resulted in less pulmonary lesion and splenic damage than antibiotic therapy alone (Safwat et al.). Essential oils isolated from *Cissampelos oppositifolia* that also contain α-pinene, δ-carene, and caryophyllene were also very synergistic with antibiotics. δ-Carene was tested as having MICs of 0.04 mg/mL against MRSA and 0.05 mg/mL against *E. coli*. Time-kill assays indicated that the bacteria were killed in 2–4 h with amoxicillin or erythromycin. FTIR/UV spectra data indicated that there were structural changes, and molecular docking showed good binding with antibiotics (Zhao et al.).

Computational studies broadened the treatment landscape by virtually screening natural products against the metallo-β-lactamase VIM-1 of *Pseudomonas aeruginosa*, which afforded 4 inhibitors (CNP0390322, CNP03905695, CNP0079056, CNP0338283) with superior docked scores and stable interactions in a one-microsecond molecular dynamics simulation, which may restore β-lactam activity (Ardawi et al.).

With the advancement of Immunotherapeutic approaches the discovery of Staphylococcal Protein A (SpA) epitopes resulted in the construction of a multi-epitope which resulted in high bindin affinities to HLA, and TLR-4 interactions which may indicate viable vaccine candidates against MRSA (Zhou et al.). Likewise, analysis of non-structural proteins of the hepatitis C virus identified 27 CTL epitopes, three of which were more than 90% conserved and exhibited significantly strong immunogenic responses after docking to HLA-A*02:01 and TLR-3, indicating they are reasonable vaccine candidates (Tudi et al.).

Finally, Shang et al. discussed how microbial natural products further demonstrated their value in antibiotic discovery. *Streptomyces kanamyceticus*, which was obtained from a soil source, exhibited antimicrobial activity with inhibition zones as great of 30 mm, with MIC values from 20 to 70 µg/mL. Maximal production of metabolites was achieved through media optimization with glucose and soybean meal at a nutrient concentration of 10 g/L. This was further evidence of the value of microbial sources for new antibiotics.

## Conclusion

Overall, these nine submissions reflect the changing paradigm of MDR therapy; moving away from antibiotics solely, towards combinative approaches that incorporate a variety of disciplines. Both PDT, nanocarriers, silver nanoparticles, and essential oil can function as immediate translational options, while bioinformatics-based inhibitor discovery and epitope-based vaccines are probable options for the future. Plant-derived extract and microbial metabolites also reiterate the value of natural products as resistance modulators. Altogether, these developments reaffirm that the future of combating MDR treatments will be deducing a combination: where a natural compound, an independently smart delivery system, and even immunology-based interventions act side-by-side, in addition to antibiotics, to outsmart evolving resistance to aid in improved global health.
